# Quantitative acetylome analysis reveals histone modifications that may predict prognosis in hepatitis B‐related hepatocellular carcinoma

**DOI:** 10.1002/ctm2.313

**Published:** 2021-03-08

**Authors:** Xiaoqiang Chai, Jianfei Guo, Ruizhao Dong, Xuan Yang, Chao Deng, Chuanyuan Wei, JiaJie Xu, Weiyu Han, Jiacheng Lu, Chao Gao, Dongmei Gao, Cheng Huang, Aiwu Ke, Shuangqi Li, Huanping Li, Yingming Tian, Zhongkai Gu, Shuxian Liu, Hang Liu, Qilong Chen, Feng Liu, Jian Zhou, Jia Fan, Guoming Shi, Feizhen Wu, Jiabin Cai

**Affiliations:** ^1^ Department of Liver Surgery and Transplantation of Zhongshan Hospital, Liver Cancer Institute of Zhongshan Hospital, Key Laboratory of Carcinogenesis and Cancer Invasion of Ministry of Education, Laboratory of epigenetics of Institutes of Biomedical Sciences, Key Laboratory of Birth Defects of Children's Hospital Fudan University Shanghai China; ^2^ Shanghai Center for Plant Stress Biology Center for Excellence in Plant Molecular Sciences Chinese Academy of Sciences Shanghai China; ^3^ School of Basic Medical Sciences Fudan University Shanghai China; ^4^ Institute of Interdisciplinary Integrative Medicine Research Shanghai University of Traditional Chinese Medicine Shanghai China; ^5^ Shenzhen Branch, Guangdong Laboratory for Lingnan Modern Agriculture, Genome Analysis Laboratory of the Ministry of Agriculture Agricultural Genomics Institute at Shenzhen Chinese Academy of Agricultural Sciences Shenzhen China

**Keywords:** hepatocellular carcinoma, histone, lysine acetylation, prognostic factor, proteomics

## Abstract

Lysine acetylation (Kac) as an important posttranslational modification of histones is essential for the regulation of gene expression in hepatocellular carcinoma (HCC). However, the atlas of whole acetylated proteins in HCC tissues and the difference in protein acetylation between normal human tissues and HCC tissues are unknown. In this report, we characterized the proteome and acetyl proteome (acetylome) profile of normal, paracancerous, and HCC liver tissues in human clinical samples by quantitative proteomics techniques. We identified 6781 acetylation sites of 2582 proteins and quantified 2492 acetylation sites of 1190 proteins in normal, paracancerous, and HCC liver tissues. Among them, 15 proteins were multiacetylated with more than 10 lysine residues. The histone acetyltransferases p300 and CBP were found to be hyperacetylated in hepatitis B virus pathway. Moreover, we found that 250 Kac sites of 214 proteins were upregulated and 662 Kac sites of 451 proteins were downregulated in HCC compared with normal liver tissues. Additionally, the acetylation levels of lysine 120 in histone H2B (H2BK120ac), lysine 18 in histone H3.3 (H3.3K18ac), and lysine 77 in histone H4 (H4K77ac) were increased in HCC. Interestingly, the higher levels of H2BK120ac, H3.3K18ac, and H4K77ac were significantly associated with worse prognosis, such as poorer survival and higher recurrence in an independent clinical cohort of HCC patients. Overall, this study lays a foundation for understanding the functions of acetylation in HCC and provides potential prognostic factors for the diagnosis and therapy of HCC.

## Graphical abstract

Our analysis showed a landscape of lysine acetylation in HCC. And we clearly identified significantly altered acetylated sites in normal, paracancerous and HCC liver tissues and validated the clinical significance of three histone acetylation sites using an independent cohort.

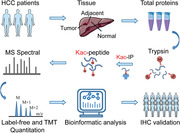

**Abbreviations**
ACN: acetonitrile
AGC: automatic gain control
BP: biological process
CC: cellular component
FA: formic acid
FC: fold change
FDR: false discovery rate
GLUD1: glutamine synthetase 1
GO: Gene Ontology
HAT: histone acetyltransferase
HBV: hepatitis B virus
HBX: HBV X protein
HCC: hepatocellular carcinoma
IHC: immunohistochemistry
Kac: lysine acetylation
KEGG: Kyoto Encyclopedia of Genes and Genomes
LIHC: Liver Hepatocellular Carcinoma
MF: molecular function
PBS: phosphate‐buffered saline
PPI: protein–protein interaction
PTM: posttranslational modification
TCA: trichloroacetic acid
TCGA: The Cancer Genome Atlas
TEAB: triethylammonium bicarbonate
TMA: tissue microarray
TMT: tandem mass tag
WB: western blotting

## 1 INTRODUCTION

Hepatocellular carcinoma (HCC) is the fourth most common tumor in the world.^1^ The occurrence and development of HCC are mainly caused by cirrhosis, hepatitis B virus (HBV), or hepatitis C virus infection. The incidence of HBV‐related HCC accounts for nearly 85% of HCC patients in China.^2^ Lysine acetylation (Kac) is a posttranslational modification (PTM) that is critical for gene expression and plays an important role in chromatin remodeling, transcription factor activity, and metabolic enzyme activity.^3^ A number of acetylation studies related to cancer have been reported. For instance, hyperacetylation of mitochondrial proteins in kidney cells affects metabolic and antioxidant processes.^4^ The acetylome in colorectal cancer exhibits differential regulation in primary and distant metastatic tumors.^5^ The acetylation of proteins in the mouse liver correlates with the circadian and feeding rhythms, and the overrepresented mitochondrial acetylated proteins were regulated by rhythms and depend on NAD^+^‐dependent SIRT3 deacetylation.^6^ However, the acetylome atlases in HCC, paracancerous, and normal liver tissues are unknown, which hampers the understanding of acetylation role in HCC pathology. Recently researches reported a tandem mass tag (TMT)‐labeling acetylome for human HCC and normal tissues,^7^ but the number of Kac proteins and sites was lower than ours. Acetyl‐CoA is the key central metabolite and the donor of the acetyl group in protein acetylation. Changes of cellular acetyl‐CoA levels regulate histone and nonhistone acetylation. For example, the acetyl‐CoA thioesterase 12 regulates acetyl‐CoA metabolism, and histone acetylation promotes HCC metastasis by epigenetic induction of epithelial–mesenchymal transition.^8^ These findings suggest that acetylation may play a critical role in HCC development and recurrence, and associate with the prognosis of HCC.

In this study, we analyzed the changes of protein acetylation level in hepatitis B‐related HCC and normal liver tissues of clinical samples using label‐free and TMT‐labeling quantification proteomics. More than 1000 acetylated lysine residues were identified, and most of them were hyperacetylated. The acetylation level of some Kac sites (such as histones) showed significant differences between HCC and normal liver tissues. Based on the western blotting (WB) and immunohistochemistry (IHC) results of an independent cohort of HCC patients, we demonstrated that lysine 120 in histone 2B (H2BK120ac), lysine 18 in histone H3.3 (H3.3K18ac), and lysine 77 in histone H4 (H4K77ac) were significantly associated with survival of HCC patients. More interestingly, the H4K77ac was associated with HCC recurrence. This indicates that H2BK120ac, H3.3K18ac, and H4K77ac may be potential prognostic factors for HCC. Our data provides a landscape of acetylation in HCC and establishes the potential of acetylation sites as prognostic factors of HCC.

## 2 MATERIALS AND METHODS

### 2.1 Patients and follow‐up

All patients involved in our research were HBV infected. Fresh tumor samples were taken from areas adjacent to the tumor margins from consecutive patients with HBV‐related HCC who underwent curative resection in 2016 at the Liver Cancer Institute, Zhongshan Hospital, Fudan University. A total of two normal liver tissues from two patients and three paired paracancerous and HCC tissues from the other three patients were used for total proteome and acetylome quantification by label‐free quantitative proteomics. Three paired paracancerous and HCC tissues from three patients were used for TMT labeling quantification (Table S1). A cohort of 135 HCC patients were randomly selected from consecutive patients who underwent curative resection from May 2012 to May 2013. Clinicopathological characteristics were defined as described previously.^9^ Ethical approval was obtained from the Research Ethics Committee of Zhongshan Hospital, Fudan University. A signed informed consent was obtained from each patient. The follow‐up data were summarized at the end of December 2018, with a median follow‐up of 59 months (range: 7–73 months). Follow‐up procedures were described in our previous study.^9^

### 2.2 Cell lines

The HCC cell line HepG2 (American Type Culture Collection) and the human liver cell line L02 (Cell Bank of the Chinese Academy of Sciences) were maintained in DMEM supplemented with 10% fetal bovine serum and 1% penicillin–streptomycin at 37°C with 5% CO_2_.

### 2.3 Protein extraction

Samples were ground into powder with liquid nitrogen,^10^ then transferred to a 5‐mL centrifuge tube. Then the samples were sonicated in lysis buffer (8 M urea, 1% Triton‐100, 65 mM dithiothreitol [DTT, Sigma], and 0.1% Protease Inhibitor Cocktail III) three times on ice using a high‐intensity ultrasonic processor (Scientz). Debris was removed by centrifugation at 20,000 × *g* at 4°C for 10 min. Finally, the proteins were precipitated with 15% cold trichloroacetic acid (TCA) for 2 h at –20°C. After centrifugation at 4°C for 10 min, the supernatant was discarded. The precipitates were washed three times with cold acetone. The protein pellets were redissolved in 8 M urea in 100 mM triethylammonium bicarbonate (TEAB) (pH 8.0) (Sigma–Aldrich, Saint Louis, USA). The protein concentration was determined with 2‐D Quant kit (GE Healthcare) according to the manufacturer's instructions.

### 2.4 Protein digestion

The proteins in 8 M urea in 100 mM TEAB buffer were reduced with 10 mM DTT for 1 h at 37°C and alkylated with 20 mM iodoacetamide (Sigma) for 45 min at room temperature in darkness. The alkylated solution was diluted by adding 100 mM TEAB until the urea concentration was below 2 M. Proteins were digested overnight with modified porcine trypsin (Promega, Madison, USA) at a protease/substrate ratio of 1:50 (w/w), followed by a second round digestion for 4 h with trypsin at a protease/substrate ratio of 1:100 (w/w). Tryptic peptides were desalted by Strata X C18 SPE column (Phenomenex) and vacuum‐dried for TMT labeling and label‐free quantification.

1 **HIGHLIGHT**

- Acetylome in normal, paracancerous, and HCC liver tissues
- HSPD1, HADHA, CPS1, GLUD1, and ADH1B were multiacetylated with more than 10 lysine sites
- Hyperacetylation of p300 and CBP in HCC compared with paracancerous tissues
- Higher levels of H2BK120ac, H3.3K18ac, and H4K77ac were associated with worse prognosis

### 2.5 TMT labeling

The lyophilized peptides were solubilized in 0.5 M TEAB and 6‐plex TMT labeling was performed according to the manufacturer's protocol of the kit (Thermo Scientific, 90068, Waltham, USA). Briefly, one unit of the TMT reagent (defined as the amount of reagent required to label 100 μg of protein) was thawed and reconstituted in acetonitrile (ACN). The peptides of different labeling were then incubated for 2 h at room temperature and pooled, desalted, and dried by vacuum centrifugation. Samples A1, A2, B1, B2, C1, and C2 were labeled by TMT reagents 126, 127,128, 129, 130, and 131, respectively.

### 2.6 High‐performance liquid chromatography fractionation

The TMT‐labeling peptides were fractionated by high‐pH reverse‐phase high‐performance liquid chromatography using an Agilent 300Extend C18 column (5 μm particles, 4.6 mm ID, 250 mm length). First, the peptides were separated into 80 fractions with a gradient ramping from 2% to 60% mobile phase B (100% ACN, conditioned with 10 mM ammonium bicarbonate [pH 10]) over 80 min. Next, small fractions were combined into eight major fractions and dried by vacuum centrifugation.

### 2.7 Enrichment of the acetylated peptides by immunoaffinity precipitation

To enrich lysine‐acetylated peptides, 3 μg TMT‐labeled peptides of each fraction and 1.5 mg label‐free peptides of each samples were dissolved in 300 μL NETN buffer (100 mM NaCl, 1 mM EDTA, 50 mM Tris‐HCl, 0.5% NP‐40, pH 8.0). The peptides were incubated with 20 μL prewashed antibody beads (cat no: PTM‐104, Jingjie PTM BioLabs, Hangzhou, China) at 4°C overnight with gentle shaking. The beads were then washed four times with NETN buffer and twice with ddH_2_O. Next, the bound peptides were eluted from the beads using 0.1% trifluoroacetic acid. Then the eluted peptides were lyophilized using a lyophilizer. Finally, the resulting peptides were desalted with C18 ZipTips (Merck Millipore, USA) according to the manufacturer's instructions.

### 2.8 LC–MS/MS analysis of the label‐free peptides

For label‐free experiments, the peptides from total protein digestion or acetylated‐peptide enrichment were dissolved in 0.1% formic acid (FA) and analyzed by online nanoAcquity ultraperformance LC (Waters, Milford, MA, USA) coupled with an Orbitrap Fusion Tribrid mass spectrometer (Thermo Fisher Scientific, Waltham, MA, USA). Nanospary was controlled by a PicoView Nanospray Source (PV550; New Objective, Woburn, MA, USA) at a spray voltage of 1.9 kV. The peptides were concentrated using a 2G‐V/MT Trap symmetry C18 column (5 μm particles, 180 μm ID × 20 mm length) at a flow rate of 5 μL/min for 3 min. The concentrated peptides were further separated on a BEH130 C18 analytical column (1.7 μm particles, 100 μm ID × 250 mm length) at a flow rate of 250 nL/min. Peptides were eluted from the analytical column using a 90 min and 3 min linear gradient of 3%–85% ACN in 0.1% FA. Data‐dependent MS/MS acquisition was performed following a full MS survey scan by Orbitrap at a resolution of 60,000 over the *m*/*z* range of 300–2000. The top 20 most intense precursor ions were subjected to MS/MS measurements. The target values of automatic gain controls (AGCs) were set as 200,000 for Orbitrap MS and 10,000 for ion‐trap MS/MS detection. The fragmentations of the selected multiply charged ions were achieved using helium gas and argon at a normalized collision energy of 35% for higher energy collisional dissociation fragmentation. Dynamic exclusion was enabled for 60 s. Singly charged or charge‐unassigned ions were excluded from MS/MS analysis. The peptides used for total proteome quantification were analyzed using the same methodology as above.

### 2.9 LC–MS/MS analysis of the TMT‐labeled peptides

For TMT labeling samples, the enriched lysine‐acetylated peptides were dissolved in 0.1% FA and concentrated using a reverse‐phase precolumn (Acclaim PepMap 100 C18; metric: 75 μm i.d. × 15 cm; particle size: 3 μm; pore size: 100 Å; type: nanoViper; cat no: 164568, Thermo Scientific). The peptides were separated using a reverse‐phase analytical column (Acclaim PepMap RSLC C18; metric: 50 μm i.d. × 15 cm; particle size: 2 μm; pore size: 100 Å; type: nanoViper; cat no: 164562, Thermo Scientific). The gradient was a linear increase from 7% to 20% solvent B (0.1% FA in 98% ACN) over 24 min, 20% to 35% for 8 min, then a linear increase to 80% over 3 min, then maintenance at 80% for the last 5 min. Flow rate was constant at 280 nL/min. The fractionated peptides were ionized using a nanospray source (NSI) and analyzed by Q Exactive™ Plus hybrid quadrupole‐Orbitrap mass spectrometer (Thermo Scientific) coupled to the UPLC system. The electrospray voltage applied was 2.0 kV. The precursor ions were detected in the Orbitrap at a resolution of 70,000. A data‐dependent procedure that alternated between one MS scan followed by 20 MS/MS scans was applied for the top 20 precursor ions above a threshold ion count of 2 × 10^4^ in the MS survey scan with 30.0 s dynamic exclusion. MS/MS fragmentation was performed using normalized collision energy fixed at 30. The daughter ions were detected in the Orbitrap at a resolution of 17,500. AGC was used to prevent overfilling of the ion trap; 5 × 10^4^ ions were accumulated for generation of MS/MS spectra. For MS scans, the *m*/*z* scan range was 350–1800. Fixed first mass was set as 100 *m*/*z*.

### 2.10 Database searches of LC–MS/MS data using MaxQuant

The resulting MS/MS data were processed using MaxQuant with integrated Andromeda search engine (v.1.4.1.2).^11^ The LC–MS/MS spectra were searched against the human proteome (UP000005640, Swiss‐Prot 20200629 release containing 75,069 sequences) concatenated with a reverse decoy database. Trypsin/P was specified as cleavage enzyme allowing up to four missed cleavages, four modifications, and five charges per peptide. Mass error was set to 10 ppm for precursor ions and 0.02 Da for fragment ions. Carbamidomethylation on Cys was specified as fixed modification and oxidation on Met, Acetylation on Lys, and acetylation on protein N‐terminal were specified as variable modifications. False discovery rate (FDR) thresholds for protein, peptide, and modification site were specified at 1%. Minimum peptide length was set as 7. These common parameters were used in label‐free proteome, label‐free acetylome, and TMT‐acetylome database searching analyses.

For acetylome identification (including label free and TMT‐6‐plex quantification), the following additional settings were used: Modified peptide score was set at >40. Only peptides with a site localization probability of >0.75 were retained. For total proteome identification, Kac was set to variable modification. Unless specified in the above common parameters, other searching parameters were set by default.

### 2.11 Protein quantification and differential expression analysis

For the label‐free proteome and acetylome datasets, the MaxQuant results were postprocessed using Perseus software platform.^12^ The finally processed datasets were used to calculate the fold change (FC) of acetylated sites or proteins among normal (N), paracancerous (P), and HCC (T) tissues. We calculated the FC of proteome and acetylome separately for paired groups (T vs. N, T vs. P, and P vs. N). The FCs with <0.5 or >2 were defined as down‐ or upregulation. The significance of FC was calculated using the two‐sided Student's *t* test. Thus, for each compared group, there are nine kinds of combination of differential change between protein expression and acetylation level. We define this as a “Nine‐Square scatterplot,” which clearly reflects the relationship between site acetylation level and corresponding protein expression. For TMT analyses, a FC of <0.77 or >1.3 with a *p*‐value <0.05 was considered as down‐ or upregulation.^13^

### 2.12 Western blotting

Logarithmically growing cells or tissue were washed twice with ice‐cold phosphate‐buffered saline (PBS) and lysed in RIRP lysis buffer (50 mM Tris‐HCl [pH 7.4], 150 mM NaCl, 1% NP‐40, 0.1% SDS) containing protease inhibitor cocktail. After sonication on ice, the cells or tissues lysates were centrifuged at 12,000 × *g* for 20 min at 4°C. The supernatants were boiled for 10 min in the presence of β‐mercaptoethanol. The proteins were fractionated on 10% sodium dodecyl sulfate‐polyacrylamide gel electrophoresis and transferred onto nitrocellulose membrane. The membrane was blocked in 5% dry milk‐TBST (10 mM Tris‐HCl [pH 7.5], 150 mM NaCl, and 0.1% Tween 20) for 1 h at 37°C. The membrane was incubated overnight with the primary antibody (anti‐H2BK120ac, PTM‐111; anti‐H3K18ac, PTM‐158; anti‐H4K77ac, PTM‐127; JingJie PTM Biolab, Hangzhou, China) at a dilution of 1:2000 (v/v) in TBST at 4°C. Next, the membrane was washed three times with TBST before incubating with the corresponding secondary antibody for 1 h at 37°C in TBST. Signals in membrane were visualized on X‐ray film using an enhanced chemiluminescence detection system.

### 2.13 Tissue microarray and IHC

The tissue microarrays (TMAs) from an independent cohort were used in our research. The TMAs were constructed using 135 paired tumor–nontumor liver tissues from the HBV‐related HCC cohort using the method described previously.^14^ In short, all cases were histologically investigated by H&E staining. Then the representative areas for detection were premarked on the paraffin blocks, away from necrotic and hemorrhagic regions. Duplicates of 1.5‐mm‐diameter cylinders from two contrastive areas, HCC tumor center and adjacent, were involved in each case, to ensure reproducibility and homogeneous staining of the tissue slides.

IHC staining for histone modification sites was performed on the TMAs as described previously.^15^, ^16^, ^17^ In brief, USP7/TRIP12 staining was simultaneously examined by two blinded, independent observers, and a consensus score was reached for each core. The staining intensity of USP7/TRIP12 was categorized into levels 0, 1, 2, and 3. The percentage of USP7/TRIP12‐positive cells was scored as 0 (0%), 1 (1%–33%), 2 (34%–66%), and 3 (67%–100%). In the case of differences between duplicate cores, the higher score of the two tissues was taken as the final score. The sum of the intensity and percentage score was used as the final staining score. The staining pattern was defined as follows: 0, negative; 1–2, weak; 3–4, moderate; and 5–6, strong. Rabbit anti‐acetyl‐histone H2B (Lys120) and anti‐acetyl‐histone H4 (Lys77) and mouse anti‐acetyl‐histone H3 (Lys18) were diluted 1:2000 in PBS containing 1% Bovine serum albumin (BSA). The incubation with the primary antibodies was performed at 4°C overnight. Nuclei were counterstained with hematoxylin. Immunostaining using the second antibodies and the signal detection were carried out using the protocol of the Ventana automated staining platform (Ventana Medical System).

### 2.14 Acetylated protein annotation

Gene Ontology (GO) annotations of the human proteins were retrieved from the UniProt‐GOA database (www.ebi.ac.uk/GOA). Protein domains of the identified Kac proteins were annotated using InterProScan (www.ebi.ac.uk/interpro).^18^, ^19^ Sequence motif was analyzed using iceLogo.^20^ The pathways enriched in the differential expression proteins were identified using Kyoto Encyclopedia of Genes and Genomes (KEGG) database by two‐tailed Fisher's exact test. The total identified proteins were used as the background. Correction for multiple hypothesis testing was conducted using standard FDR control methods. A *p*‐value of <0.05 was considered significant. These pathways were classified into hierarchical categories according to the KEGG database information. Similarly, a two‐tailed Fisher's exact test was employed to evaluate the significance of each domain identified. GO enrichment and KEGG pathway analysis of publicly available transcriptome data were performed using the clusterProfiler package in R (3.5.0).^21^ We also analyzed the transcriptome in HCC patient using TCGA (The Cancer Genome Atlas, https://www.cancer.gov/tcga) databases through University of California Santa Cruz (UCSC) Xena Public Data Hub (xena.ucsc.edu).^22^ Survival analysis was performed using the survival package in R (3.5.0).

The protein–protein interaction networks of the identified Kac proteins were analyzed using the STRING database (http://string-db.org/).^23^ The threshold of interaction confidence was set as 0.7. The interaction network was visualized using Cytoscape (version 3.6.1) and stringApp.^24^, ^25^ Protein structures from the Protein Data Base were analyzed by pymol software.^26^

### 2.15 Statistical analyses

The nonparametric Wilcoxon's signed‐rank test was applied to evaluate differential expression between paired samples. Independence of the data was tested using a two‐tailed Fisher's exact test and the chi‐squared test.^27^ The *p* < 0.05 was considered as significant. All statistical tests were performed in R (3.5.0).

## 3 RESULTS

### 3.1 Profiles of Kac proteins and sites of the normal, paracancerous and HCC liver tissues

**FIGURE 1:**
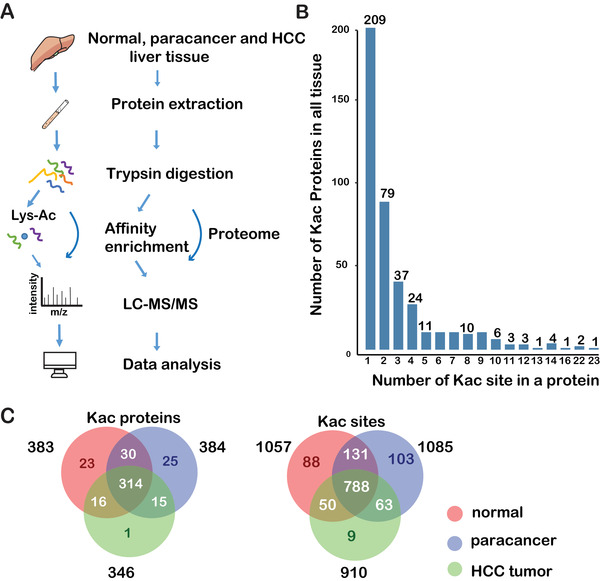
Flowchart and distribution of lysine acetylation sites and proteins in liver tissues. (A) The strategy for label‐free quantification of the proteome and acetylome of normal, paracancerous, and HCC tissues. (B) The distribution of the identified acetylated sites of acetylation proteins. (C) Venn diagram of acetylation proteins and sites in all tissues. Abbreviation: Kac, lysine acetylation

To investigate the Kac atlas of HCC, the lysine acetyl proteomics (acetylome) of hepatitis B‐related HCCs, paracancerous tissues, and normal liver tissues were analyzed using label‐free quantitative proteomic techniques (Figure 1A). A total of 1232 Kac sites from 424 proteins were identified and quantified in label‐free proteomic analysis (Table S2). Most Kac proteins contained one or two lysine residues, whereas 21 Kac proteins had ≥10 Kac sites (Figure 1B). To understand the significance of acetylation in HCC tissues, we also compared the changes of protein levels in hepatitis B‐related HCC, paracancerous, and normal liver tissues. (Tables S3 and S4). Among the 424 quantified Kac proteins, HSPD1, HADHA, CPS1, GLUD1, and ADH1B were multiacetylated (Table 1; Figures S1A and S1C). The acetylation levels of several histones, including histone H2B type 1‐K, H3.3, and H4, were increased in HCC tissues (Figures S1B and S1D). In addition, 1057 lysine residues in 383 proteins were found to be acetylated in normal liver tissue samples. There were 88 (8.3%) unique Kac sites in normal liver tissue samples (Figure 1C). There are acetylated proteins in common among the three tissues, but differ in acetylation abundance. This result indicates that these differential Kac proteins may be associated with the pathophysiological process of HCC.

**TABLE 1:** Top five multiacetylated proteins in the label‐free acetylome

Protein	N	P	T	Total	Description	Gene
P10809	19	22	21	23	60 kDa heat shock protein, mitochondrial	*HSPD1*
P40939	18	19	16	22	Trifunctional enzyme subunit alpha	*HADHA*
P31327	14	21	17	22	Carbamoyl‐phosphate synthase [ammonia]	*CPS1*
P00367	14	14	11	16	Glutamate dehydrogenase 1, mitochondrial	*GLUD1*
P00325	12	11	8	14	All‐trans‐retinol dehydrogenase [NAD(+)]	*ADH1B*

Abbreviations: N, normal; P, paracancerous; T, HCC tissue.

**FIGURE 2:**
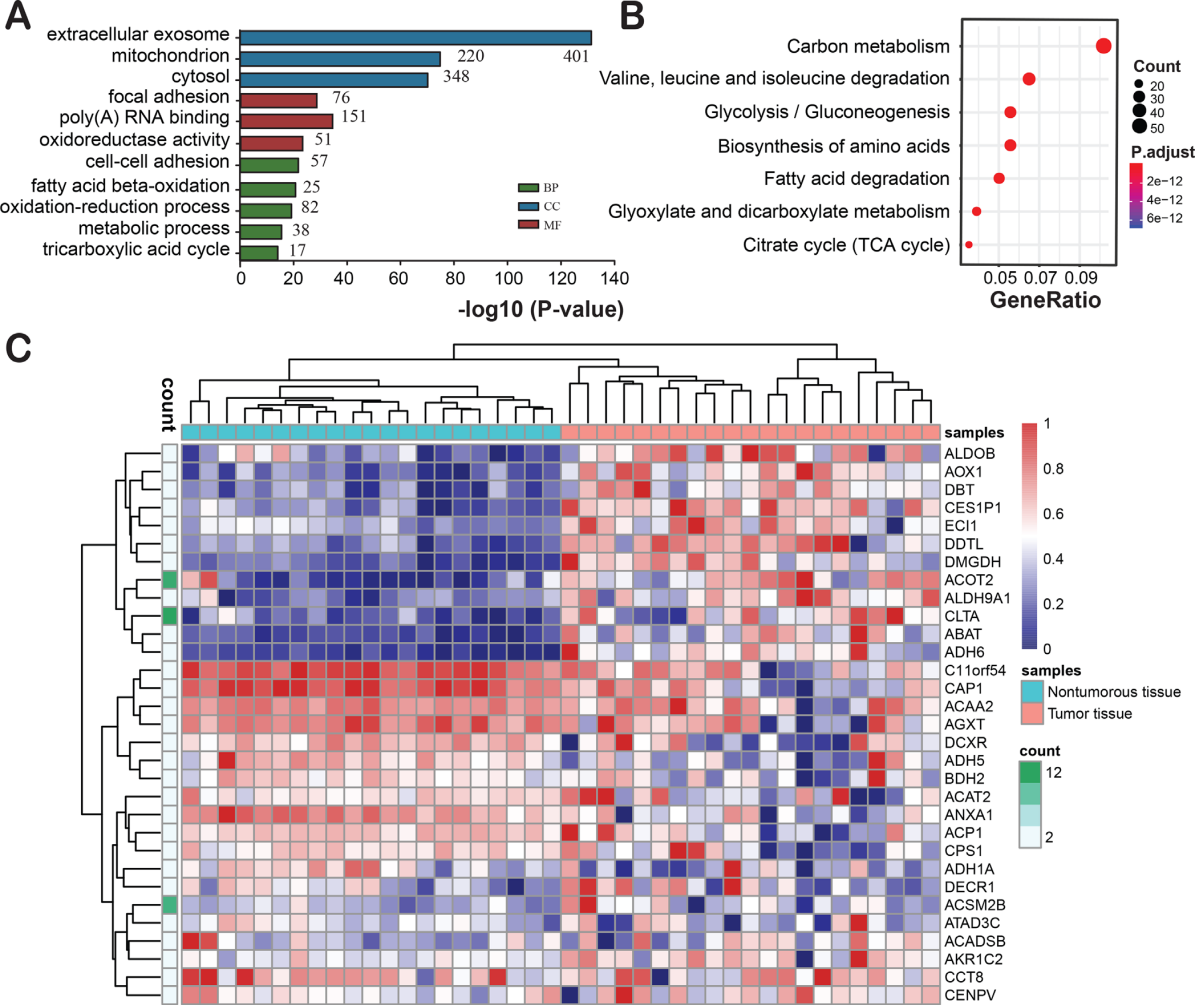
GO enrichment (A) and KEGG pathway (B) analyses of the Kac proteins in liver tissues. (C) The transcript expression level of the Kac proteins in HCC and adjacent tissues. Abbreviations: BP, biological processes; CC, cellular component; MF, molecular function

To analyze the functions or cellular distribution of the 424 Kac proteins, we performed the biological process (BP), molecular function (MF), cellular component (CC), and KEGG pathway enrichment analyses based on the GO annotation (Figure 2). BP enrichment analysis revealed that these Kac proteins were enriched in cell–cell adhesion, fatty acid beta‐oxidation, oxidation–reduction process, metabolic process, and tricarboxylic acid cycle (Figure 2A). MF analysis showed that these Kac proteins were associated with poly(A) RNA binding, focal adhesion, and oxidoreductase activity. CC analysis indicated that these Kac proteins were mainly localized in extracellular exosomes, mitochondria, and cytosol. KEGG pathway analysis suggested that these Kac proteins were significantly associated with liver metabolism‐related pathways, including “carbon metabolism,” “valine, leucine, and isoleucine degradation,” “Glycolysis/Gluconeogenesis,” “Biosynthesis of amino acids,” “Fatty acid degradation,” “Glyoxylate and dicarboxylate metabolism,” and “Citrate cycle” (Figure 2B). By analyzing a published transcriptome dataset, we found that the expression pattern of these Kac protein coding genes had different expression pattern between HBV‐related nontumorous and tumorous tissue (Figure 2C). This indicates that the Kac may contribute to the alteration of the pathophysiological process in HCC.

### 3.2 Characteristic of Kac sites and subcellular localization of Kac proteins

**FIGURE 3:**
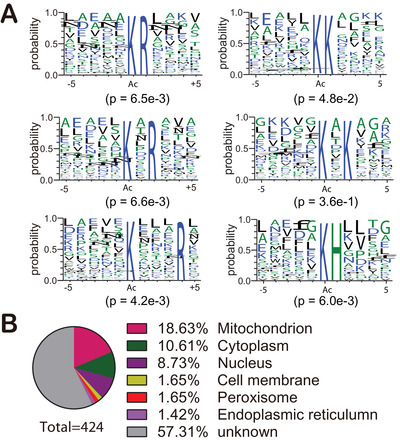
Characteristics of the identified Kac proteins. (A) Analysis of Kac motifs from positions –5 to +5 (the Kac sites were set as zero) of identified Kac proteins in label‐free acetylome. (B) Subcellular localization prediction of Kac proteins in liver tissues

To understand the sequence characteristics around the Kac sites, we analyzed the relative frequency of the 10 amino acid residues at the N‐ and C‐terminus of the identified Kac sites using iceLogo. We identified six overrepresented motifs surrounding the Kac sites, including KK/R/H, KxK/R, and KxxxR (underlined lysine is acetylated) (Figure 3A). The analysis of all the identified Kac sites revealed that acetylation occurs in regions enriched in polar amino acids, specifically basic amino acids (K, H, and R), surrounding the lysine residue. K was in the +1 and +2 positions, H in the +1, and R in the +1, +2, and +3. These findings are consistent with the *Streptococcus pneumoniae* acetylome.^28^ Acetylation motifs of KK, KxK, and KxxK were observed in rat tissues, whereas motifs of KK/R were overrepresented in human sperm.^29^ We inferred that motifs of KK, KxK, and KxxK are to a certain extent evolutionarily conserved in different organisms.

Enzyme activity is regulated by the surrounding environment. Kac is a reversible posttranslational modification that is regulated by lysine acetyltransferases with acetyl‐CoA as a cofactor and deacetylases with NAD^+^ or Zn^2+^ as cofactors.^30^ The acetyl‐CoA level of different organelles is changeable and dynamic.^31^ Therefore, we predicted the subcellular localization of the identified Kac proteins. We found that around 38% of Kac proteins were localized in the mitochondria (18.6 %), cytoplasm (10.6 %), and nucleus (8.7 %) (Figure 3B). Compared with the nucleus, there are twice as many Kac proteins in the mitochondria than in the nucleus. Indeed, the proportion of Kac proteins in the cytoplasm and mitochondria is more than threefold higher than in nucleus (Figure 3B). This observation is consistent with the mouse liver acetylome, in which most Kac proteins located in the cytoplasm and mitochondria.^6^ Less than 2% of acetylated proteins were localized to the Golgi and peroxisomes.

### 3.3 Analysis of the differentially acetylated sites and proteins

The samples used in proteome and acetylome analyses contain three groups: normal (N), paracancerous (P), and HCC (T). To understand the function of acetylation in HCC development, we compared the differential acetylation levels between three group pairs (T vs. N, T vs. P, and P vs. N). The differential changes of acetylation were normalized against the total protein expression levels. FC was used as a criterion to calculate the differences among these three groups (Table 2). All results were listed in Table S5.

**TABLE 2:** Statistics of the differential expressed Kac sites and proteins

	Type	T vs. N	T vs. P	P vs. N
Down	Kac site	662	252	815
	Kac protein	452	202	546
Up	Kac site	250	485	85
	Kac protein	215	296	58

**FIGURE 4:**
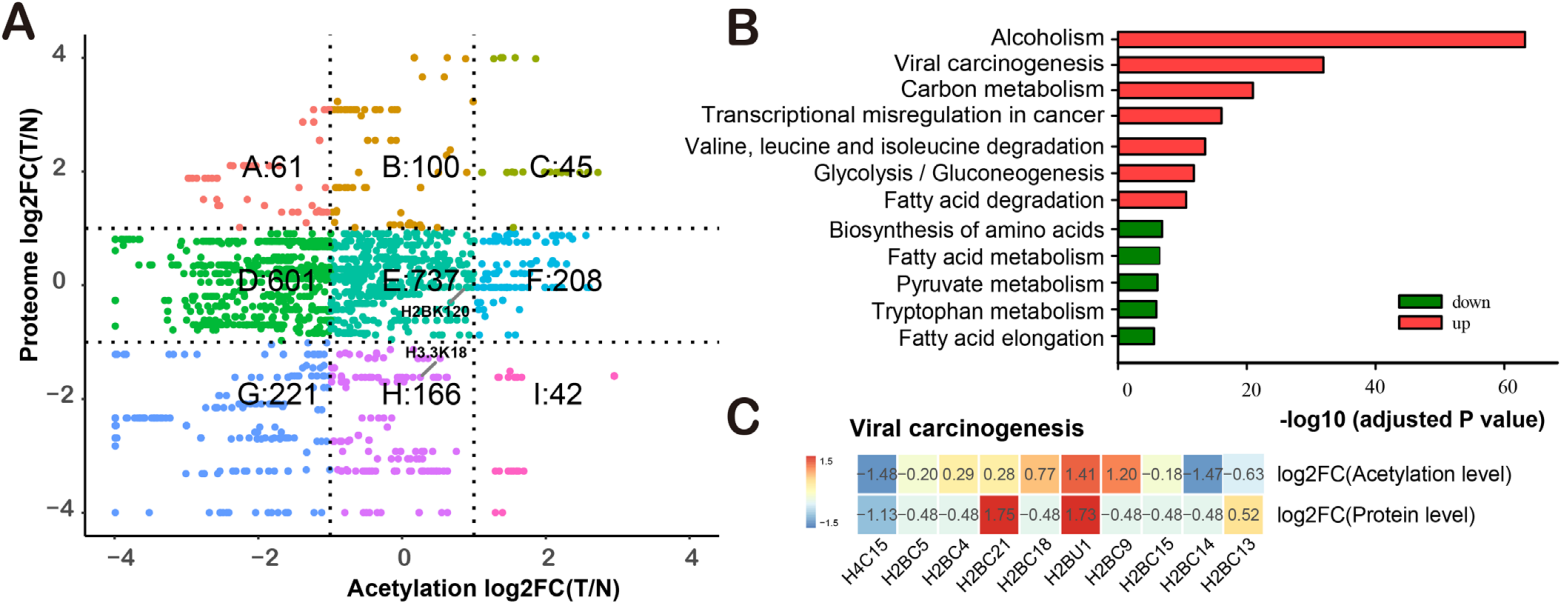
Analysis of the differential level of proteins and acetylation between the normal and HCC liver tissues. (A) The nine‐quadrant scatterplot for fold changes (T vs. N) of proteins and acetylation sites. (B) KEGG pathway enrichment analysis of the differential Kac proteins between normal and HCC liver tissues. (C) Fold changes of indicated proteins and Kac proteins between normal and HCC liver tissues in viral carcinogenesis pathway. Abbreviations: T, tumor; N, normal

We performed “Nine‐Square scatterplot” analysis of the label‐free quantitative proteome and acetylome data. The proteins were divided into nine groups (Figure 4A). Based on the strictly filtered upregulated (F and I) and downregulated (A and D) acetylation groups, we found that 250 Kac sites in 215 proteins were upregulated (FC > 2) and 662 Kac sites in 452 proteins were downregulated (FC ← 2) in HCC tissues versus normal liver tissues (Table 2; Figure 4A). GO enrichment analysis indicates that downregulated Kac proteins are enriched in small molecule catabolic and metabolic process, such as carboxylic acid catabolic process, organic acid catabolic process, drug metabolic process, and cellular amino acid catabolic process (Figure S2A), whereas the upregulated Kac proteins are mainly enriched in establishment of organelle localization, intracellular transport, RNA processing, apoptotic process, negative regulation of cellular process, and some positive regulation of macromolecule biosynthetic process, such as RNA metabolic process, nucleic acid metabolic process, and ribonucleoprotein complex biogenesis (Figure S2B). Lysine is a positive‐charge amino acid and acetylation of this residue neutralizes its electric charge.^30^ Thus, acetylation may influence protein structure and alter metabolic enzyme activity. Researchers have found that acetylation in protein could neutralize the positive charge of lysine residue, change the protein conformation, impacts protein activity,^32^ and result in metabolism regulation.^33^ The KEGG pathway analysis showed that the Kac proteins with upregulated acetylation level on their sites in HCC are mainly involved in alcoholism, viral carcinogenesis, transcriptional misregulation in cancer, glycolysis/gluconeogenesis, and fatty acid degradation (Figure 4B), whereas the proteins with downregulated acetylation levels are primarily involved in biosynthesis of amino acids, fatty acid metabolism, pyruvate metabolism, and tryptophan metabolism. The level of some proteins and Kac proteins in the viral carcinogenesis pathway (hsa05203 in KEGG database) was significantly upregulated (Figure 4C). One of the Kac protein is H2B, a core component of the nucleosome that is highly expressed in HCC tissue.^34^ H2B is also involved in disease pathway regulation in alcoholism, viral carcinogenesis, and systemic lupus erythematous.^35^

The comparisons of T versus P and P versus N groups were performed with a similar approach as above (Figures S3 and S4). We found that the increase or decrease in acetylation level of most proteins was independent of protein levels (Figures 4A, S3, and S4). That is, the increase or decrease in Kac proteins was due to changes in acetylation levels rather than changes in protein levels. The comparisons of T versus P with the TMT‐labeling approach in other three paired samples also showed similar pattern in each group (Table S6). A total of 232 Kac sites in 228 Kac proteins were simultaneously quantified in the label‐free and TMT‐labeling acetylome (Figure S5). The BPs and KEGG pathways of the up‐ and downregulated Kac proteins in each group were also highly reproducible (Figures 4, S3, and S4), further demonstrating the accuracy of the identified acetylome data.

### 3.4 Subcellular localization of the differential Kac proteins in HCC and normal liver tissues

**FIGURE 5:**
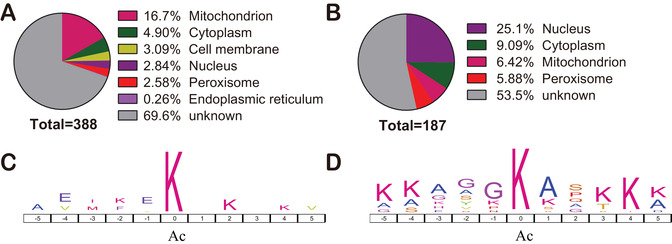
Subcellular localization and motif analyses of the differential Kac proteins between normal and HCC liver tissues. Subcellular localization (A) and motif (C) of the Kac proteins with downregulation acetylation level. Subcellular localization (B) and motif (D) of the Kac proteins with upregulation acetylation level

To know the cellular compartment of differentially acetylated proteins, we predicted the subcellular localization of the up‐ and downregulated Kac proteins in HCC. The upregulated Kac proteins in HCC compared with normal liver localize in cytoplasm (25%) and nucleus (9%), whereas downregulated Kac proteins localize in mitochondria (17%) and cytoplasm (5%) (Figures 5A and 5B). Motif analysis of the up‐ and down‐regulated Kac sites revealed that Lys (K) was overrepresented at –5, –4, +1, +3, +4, and +5 position. Lys (K) was overrepresented at +2 and +4 position of the downregulated Kac sites (Figure 5C), whereas Gly (G) was overrepresented at –3, –2, and –1 position of the upregulated Kac proteins (Figure 5D). A previous report found that acetylation of glyceraldehyde‐3‐phosphate dehydrogenase induces its translocation from cytoplasm to nucleus.^36^ Thus, we speculate that acetylation of these differential Kac proteins may impact protein localization.

### 3.5 Acetylation of histone acetyltransferases in HCC

Histone acetyltransferases (HATs) are enzymes that can transfer acetyl groups to lysine residues of histones.^37^ HATs can be autoacetylated or acetylated by other HATs.^38^ Two important HATs, EP300 and CBP, were also found to be acetylated in HCC tissue (Table S6). As the bifunctional enzymes, p300 and CBP acetylate histones and act as transcriptional coactivators that regulate cell proliferation and differentiation.^39^, ^40^, ^41^ Thus, the acetylation of p300 and CBP in HCC may alter its activity and regulate oncogene expression.^42^, ^43^, ^44^ However, the significance of the hyperacetylation of p300 and CBP remains unknown.

### 3.6 Protein–protein networks of acetylation proteins

**FIGURE 6:**
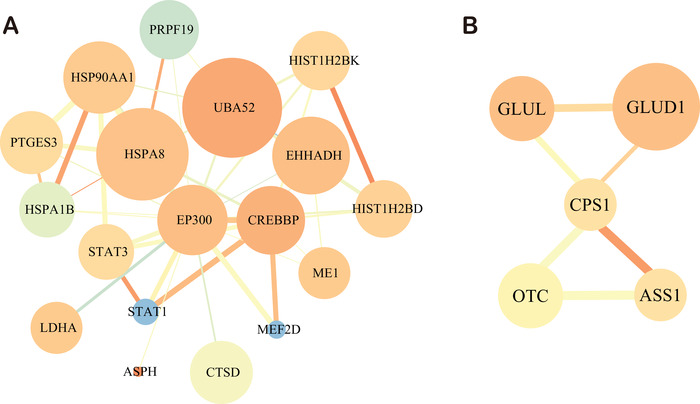
Protein–protein interaction network of Kac proteins. (A) The sub network of p300 and its interacting Kac proteins. (B) The subnetwork of CPS1 and its interacted Kac proteins. The size of node denotes the degree that interact with other Kac proteins. The color of node ranges from saffron yellow to blue, which refers to the connected clustering coefficient with corresponding Kac proteins. The thickness of edge size indicates the strength associated with corresponding Kac proteins. The color of edge ranges from saffron yellow to blue, which refers to the degree of edge betweenness

**FIGURE 7:**
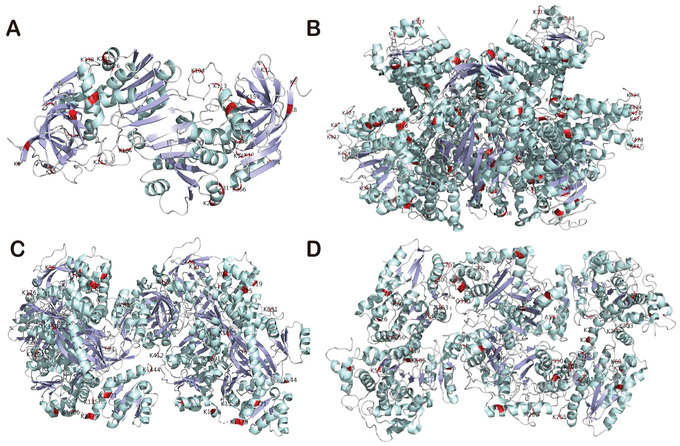
The crystal structures of four multiacetylated proteins: ADH1B (A), GLUD1 (B), CPS1 (C), and HADHA (D). Acetylated sites are labeled in red. Pale green and light blue denote α helixes and β sheets, respectively. Red color represents the position of acetylated lysine (K) sites

Protein–protein interaction (PPI) plays important roles in a variety of BPs, such as signal transduction and energy metabolism. We performed PPI network analysis of the acetylome in HCC tissues using STRING database (Figure S6). We found that EP300 and CPS1 interact with multiple Kac proteins (Figure 6). For instance, histone acetyltransferase EP300 strongly correlated with the upregulated Kac protein CREBBP (Figure 6A). CPS1 interacted with the multiacetylated glutamine synthetase 1 (GLUD1), the downregulated Kac protein argininosuccinate synthase 1, and ornithine carbamoyltransferase (Figure 6B). To understand the role of the Kac sites in proteins, pymol software was used to visualize the structures of ADH1B, GLUD1, CPS1, and HADHA with Kac sites (Figure 7). We found that Kac sites in these four proteins are more likely to target ordered secondary structure, such as alpha helices, beta‐sheets, and turns, which is consistent with previous report.^45^ In addition to the known Kac sites in UniProt^46^ and PhosphoSitePlus,^46^, ^47^ we also identified novel Kac sites in these Kac proteins including K72, K89, K301, K405, and K418 in HSPD1; K6, K9, K19, K20, K33, K105, K114, K160, K227, K232, and K355 in ADH1B; K200 and K397 in GLUD1; K138, K176, K253, K905, and K1498 in CPS1; and K213, K230, K255, K292, and K414 in HADHA. The Kac sites of ADH1B, GLUD1, CPS1, and HADHA in this dataset were also labeled in the three‐dimensional structures (Figure 7). Besides, most of the loci can also be found in public databases, which further demonstrates the accuracy of our acetylome data.

### 3.7 Kac sites of histones are potential prognostic factors for HCC

**FIGURE 8:**
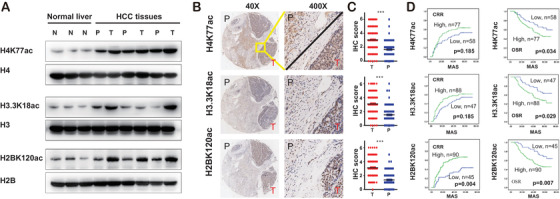
Histone acetylation is associated with prognostic survival of HCC patients. (A) Western blotting analysis of H2BK120ac, H3.3K18ac, and H4K77ac in normal, paracancerous, and HCC tissues. Immunohistochemistry (IHC) staining (B) and IHC scores (C) of H2BK120ac, H3.3K18ac, and H4K77ac in paracancerous and HCC tissues. (D) Correlation of H2BK120, H3.3K18, and H4K77 acetylation level with overall survival and recurrence. Abbreviations: CRR, cumulative recurrence rates; MAS, months after surgery; N, normal; OSR, overall survival rates; P, paracancerous; T, tumor

To understand the potential clinical significance of differential Kac proteins and sites in HCC, we investigated the acetylation level changes of the histone Kac sites in HCC and normal liver tissues. We found that the acetylation level of lysine 120 on histone H2B (H2BK120ac), lysine 18 on histone H3.3 (H3.3K18ac), and lysine 77 on histone H4 (H4K77ac) was upregulated in HCCs compared with paracancerous or normal liver tissues (Figure 4A; Table S6). WB also showed the same results in normal and HCC liver tissues (Figure 8A). IHC staining confirmed that H2BK120ac, H3.3K18ac, and H4K77ac were highly present in tumor tissues (Figures 8B and 8C). In addition, these three Kac sites significantly were associated with disease features in an independent cohort containing 135 HCC patients in a TMA (Tables 3 and S7; Figure 8D). High level of H2BK120ac was associated with poor differentiation (*p* = 0.002), whereas level of H3.3K18ac was related to microvascular invasion (*p* = 0.031). High level of H4K77ac was correlated with elevated alpha‐fetoprotein (*p* = 0.035), larger tumors (*p* = 0.017), and microvascular invasion (*p* = 0.047). Patients with high acetylation levels of all three histone Kac types showed obviously poorer overall survival than patients with low acetylation levels. Intriguingly, patients with high acetylation level of H4K77ac showed significantly shorter disease‐free survival than patient with low acetylation level (Figure 8D). We performed an analysis of The Cancer Genome Atlas Liver Hepatocellular Carcinoma (TCGA‐LIHC) dataset using GEPIA2.^34^ We found that the gene expression levels of the multiacetylated proteins HSPD1, CPS1, and ADH1B are correlated with improved survival of HCC patients, whereas the multiacetylated proteins HADHA and GLUD1 are not (Figure S7A–E). The gene expression levels of HIST1H2BC, HIST1H2BK, H3F3A, and HIST1H4A are also not related to survival.^34^ In addition, we analyzed the relationship between the expression level of the Kac protein coding genes and the overall patient survival rate by calculating the counts of acetylated (Ac) and nonacetylated (non‐Ac) protein genes in the significant (*p*‐value of odds ratio between Ac and non‐AC was used to determine significance) and nonsignificant groups using TCGA‐LIHC cohort data. The results show that the acetylation tends to target proteins that are significantly associated with overall survival (Figure S7F). Together, these data indicate that H2BK120ac, H3.3K18ac, and H4K77ac may be potential prognostic factors for HCC.

**TABLE 3:** Histone acetylation level was associated with tumor feature

	ac‐H2BK120		ac‐H3.3K18		ac‐H4K77	
	*p*	OR	*p*	OR	*p*	OR
Tumor differentiation	0.002	3.989362	–		–	
Microvascular invasion	–		0.031	0.4231343	0.047	0.4427245
AFP	–		–		0.035	2.275
Tumor size	–		–		0.017	2.573427

Abbreviations: AFP, α‐fetoprotein; OR, odds ratio; *p*, *p*‐value.

## 4 DISCUSSION

Kac plays a critical role in gene expression in HCC.^48^, ^49^ In the current study, we analyzed the acetylomes and proteomes of normal liver, paracancerous, and HCC tissues using label‐free quantitative LC–MS/MS. In total, we identified 6781 Kac sites in 2,582 Kac proteins. When only the leading proteins in MaxQuant results are considered, 1232 Kac sites in 424 proteins were quantified. After normalizing against the identified total proteins, 250 upregulated Kac sites of 214 Kac proteins and 662 downregulated Kac site of 451 Kac proteins were identified in the HCC tissues compared with the normal liver tissues. We also analyzed the acetylomes of the paracancerous and HCC tissues using TMT‐labeling proteomics technique and identified 1040 Kac sites in 587 Kac proteins. In this analysis, 237 up‐ and 122 downregulated Kac proteins of more than ±1.3 FC were identified in HCC tissues comparing with paracancerous tissues.

In the TMT quantitative proteomics analysis, the acetylation of the acetyltransferases CBP and p300 was significantly increased in HCC versus paracancerous tissue. The enrichment analysis of the differential Kac proteins between pairs of groups (T vs. N, T vs. P, and P vs. N) shows that the up‐ and downregulated Kac proteins share some common signaling pathways, such as viral carcinogenesis, glycolysis/gluconeogenesis, and fatty acid metabolism. This suggested that the protein acetylation may play an important role during HBV infection. Interestingly, H2BK120ac, H3.3K18ac, and H4K77ac are negatively correlated with survival of HCC patients, and H4K77ac is associated with HCC recurrence. Together, these results show the first landscape for HCC acetylome, and demonstrate that the H2BK120ac, H3.3K18ac, and H4K77ac may be the potential prognostic factors for HCC.

Although the characterizations and functions of acetylation in histone or nonhistone in HCC remain to be determined, five proteins including HSPD1, HADHA, CPS1, GLUD1, and ADH1B were multiacetylated in more than 14 Kac sites (Table 1). The protein and acetylation levels of these multiacetylated proteins in proteome and acetylome showed diverse patterns in normal, paracancerous, and HCC tissues (Figures S1A and S1C). Twenty‐three lysine residues in HSPD1 were found to be acetylated. HSPD1 is a 60 kDa mitochondrial heat shock protein involved in mitochondrial protein import and macromolecular assembly^50^ and the regulation of HBV viral proteins.^51^ CPS1 is a mitochondrial protein and a prognostic marker of liver cancer. The function of CPS1 is to remove excess ammonia from the cell.^52^, ^53^ In our datasets, 16 lysine residues of CPS1 were found to be acetylated, especially the Kac sites within carbamoyl‐phosphate synthetase large subunit‐like domain and ATP‐binding domain.^54^ Moreover, CPS1 is preferentially expressed in liver tissue.^55^ Thus, the highly expressed and acetylated CPS1 in liver cells may be involved in development of liver cancer. HADHA is a mitochondrial trifunctional enzyme specifically involved in the last three of the four reactions of long‐chain fatty acids beta‐oxidation pathway,^56^, ^57^, ^58^ and the acetylation levels of the K60, K129, and K569 of HDAHA were also downregulated in tumor tissues,^7^ which is consistent with our results (Table S6). Moreover, several novel Kac sites were identified in this study (Figure 7D). GLUD1 is a mitochondrial protein that participates in the anaplerosis of glutamine and produces alpha‐ketoglutarate, which is an important intermediate in TCA cycle^59^ and amino acid metabolism.^60^ In their work, Zhao et al. also found that GLUD1 was acetylated at K191, K390, K457, K489, and K527 and all the acetylation levels were downregulated in tumor tissues,^7^ which is consistent with our data (Table S6) ADH1B is a disease‐associated protein of alcohol dependence and fetal alcohol syndrome,^61^ which catalyzes the NAD‐dependent oxidation of all‐trans‐retinol and its derivatives involved in retinoid metabolism.^58^, ^62^ These results indicate that this multiacetylated protein may be involved in the response to proliferation, migration, and invasion in HCC.

A previous report demonstrated that the subcellular distribution of the Kac proteins is dramatically different from the phosphorylated proteins, and the number of phosphorylated proteins in mitochondria is threefold less than the Kac proteins that are enriched in muscle contraction and involved in ATP generation.^63^ Some oncogenic signaling‐associated proteins contain Kac sites in their functional domain, which might regulate protein localization. For instance, CNK1 is involved in cell proliferation and migration and this protein contains an acetylation site within its pleckstrin homology domain that drives its localization to the membrane.^64^ Taken together, the Kac proteins are mainly localized in cytosol, nucleus, and mitochondria, and these Kac proteins may play important roles in HCC occurrence.

The GO term and KEGG pathway enrichment analysis indicate that the upregulated Kac proteins of HCC are significantly enriched in the pentose phosphate pathway, antigen processing and presentation, and viral carcinogenesis. Conversely, downregulated Kac proteins in HCC were mainly enriched in lysine degradation, fatty acid degradation, and metabolism of xenobiotics by cytochrome P450. As revealed by the TMT‐labeling data, the acetylation level of the highly upregulated Kac protein GSTO1 in HCC tissues is more than 10 times that of nontumor tissues. GSTO1 is a member of GSTs family that catalyzes the conjugation of glutathione to distinct endogenous and exogenous compounds that are involved in different BP.^65^ Variants of GSTs affect enzyme activity and may influence individual susceptibility to cancer.^66^ The high degree of hyperacetylation of GSTO1 at K198 may affect its enzyme activity, because the K198 and E154 are located in the same pocket of the GSTO1 structure and are opposite to each other.^67^ A deletion of E154 in GSTO1 exhibits refolding defects and low stability, resulting in GSTO1 deficiency.^67^ Therefore, we speculate that hyperacetylated GSTO1 might increase the risk of HCC.

**FIGURE 9:**
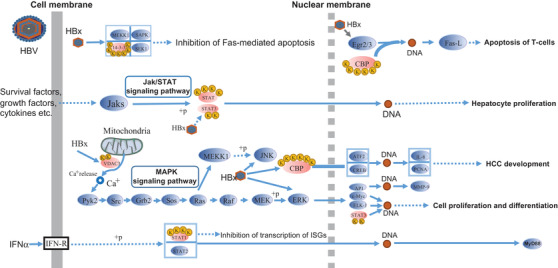
The diagram of proteins with upregulated acetylation between HCC and paracancerous tissues in hepatitis B virus pathway (extracted from KEGG database, hsa05161). The yellow circle with K (lysine) represents acetylated lysine residues. The ellipse with light red and light blue color refers to Kac proteins with upregulated acetylation level and proteins without lysine acetylation in hepatitis B virus pathway, respectively. The solid and dotted gray vertical lines indicate the cell membrane and nuclear membrane, respectively

HBV infection stimulates carcinogenesis.^68^, ^69^, ^70^ We found that Kac proteins were enriched in viral carcinogenesis (Figures 4B and S4B). We analyzed the Kac proteins of HBV pathway using TMT‐labeling quantitative data. We found that CBP (CREBBP), EP300, STAT3, VDAC3, 14‐3‐3ζ (YWHAZ and YWHAB), and STAT1 were hyperacetylated (Figure 9). CBP may interfere with the regulation of HBV pathway. A previous study found that STAT3 and STAT1 acetylation might be involved in hepatocyte proliferation in the Jak/STAT signaling pathway.^71^ HBV X protein (HBX) is a viral protein encoded by a pregenomic RNA, which regulates VDAC3 activity.^72^, ^73^ VDAC3 is a voltage‐dependent anion channel that regulates Ca^2+^ release and homeostasis.^74^ 14‐3‐3ζ are phosphorylated binding proteins, which are overexpressed in cancerous tissues of patients with HCC.^75^ We found that 12 Kac sites of 14‐3‐3ζ were identified and quantified in HCC. The acetylation may affect the interaction between 14‐3‐3ζ and HBV protein X.^75^ We found seven lysine residues in 14‐3‐3ζ were acetylated in functional domain. These residues include the K3 and K9 in the N‐terminal; the K49, K74, and K120 in the 14‐3‐3ζ binding pocket; and the K138 and K157 in an alpha helix that helps form the phosphor‐binding pocket.^76^ Thus, acetylation may regulate some key proteins of HBV pathway that is involved in HCC regulation.

Analysis of the Kac sites of histone revealed that about 20 lysine residues were acetylated (Figure S1). Histones are the core components of nucleosome and the basic unit of chromosome. The posttranslational modifications of histone are also required for gene transcription. The acetylation of lysine in histones can neutralize the positive charge of lysine, thereby reducing the accessibility of histones to DNA for gene transcription.^77^ Most of them are well investigated in liver cancer including the novel Kac sites K120 in histone H2B type 1‐C/E/F/G/I (HIST1H2BC); the K77 in histone H4 (HIST1H4A); the K303 in core histone macro‐H2A.1 (H2AFY); and the K108 and K120 in histone H2B type 1‐K (HIST1H2BK).^78^, ^79^, ^80^ Among these Kac sites, HIST1H2BC can also be hydroxyisobutyrylated, hydroxybutyrylated, and succinylated.

WB and IHC experiments demonstrated that H2BK120ac, H3.3K18ac, and H4K77ac correlate with HCC occurrence (Figure 8). For instance, H2BK120ac is correlated with poor differentiation (*p* = 0.002), whereas H3.3K18ac is related with microvascular invasion (*p* = 0.031), H4K77ac is correlated with elevated alpha‐fetoprotein (*p* = 0.035), larger tumors (*p* = 0.017), and microvascular invasion (*p* = 0.047). Interestingly, H4K77ac is associated with survival and recurrence. Although H4K77ac has obvious clinical significance, there are few cancer‐related researches of H4K77ac. Researches have provided the evidence that H4K77ac may decrease DNA accessibility, leading to chromatin organization repression.^81^ The results of H3K18ac in HCC cell line HepG2 and healthy liver cell line L02 are consistent with previous studies.^82^, ^83^ Some studies also show that H3K18ac promotes the development of various cancers, such as breast, colon, lung, hepatocellular, pancreatic, prostate, and thyroid cancer.^84^ Amamoto et al. reported that H2BK120 acetylation and malonylation affect chromatin structure assembly by reducing internucleosome interactions.^85^ These results demonstrated that histone acetylation is widely involved in distinct cancers. Moreover, the analysis of the independent cohort of HCC patients further demonstrated that these three histones Kac sites are correlated with survival. Thus, we speculate that H2BK120ac, H3.3K18ac, and H4K77ac may be potential prognostic factors of HCC and are beneficial to the management of HCC patients.

With the rapid development of epigenetic studies of liver cancer, researchers have found that the epigenetic modifications are closely related to the development of cancer. The NAD^+^‐dependent HDAC (histone deacetylase) SIRT1 can regulate bile acid metabolism. SIRT1 is upregulated in HCC and promotes liver cancer by stimulating deactylation of the farnesoid X receptor to an extent that in turn dysregulates bile acid homeostasis.^86^ In addition, researchers have demonstrated that inhibition of HDAC1/2 was able to suppress proliferation and induce tumor cell death in several HCC cell lines.^87^ HBX also alters DNA methylation via its ability to affect DNMT activity, and also recruits the histone acetyltransferases (HAT) p300/CBP to induce IL‐8 and proliferating cell nuclear antigen that are involved in inflammation and cell proliferation, respectively.^88^ In summary, our study provides an overview of HCC Kac, and clarifies the acetylation changes of Kac sites and proteins in HCC and paracancerous tissues, and explores the clinical significance of H2BK120ac, H3.3K18ac, and H4K77ac in an independent cohort of HCC patients. Thus, we propose that Kac plays an important role in the occurrence and development of HCC, which may be a potential prognostic factor of HCC.

## ETHICS STATEMENT

All patients recruited into our study gave written informed consent prior to inclusion. The study was approved by the Research Ethics Committee of Zhongshan Hospital, Fudan University, Shanghai, China.

## CONFLICT OF INTEREST

The authors declare no conflict of interest.

## AUTHOR CONTRIBUTIONS

JC, FW, and SG conceived the project. The data analysis was done by XC, JF, RD, XY, and CD. FL, JZ, and JF participated in experimental design. CW, JX, WH, JL, CG, DG, CH, and AK participated in collection of the published datasets. CW, SL, HL, and YT participated in IHC and WB experiment. ZG, SL, HL, QC, and FL gave some suggestions about the manuscript writing. All authors read and approved the final manuscript.

## Supporting information

Supporting Information

Supporting Information

Supporting Information

Supporting Information

Supporting Information

Supporting Information

Supporting Information

Supporting Information

Supporting Information

Supporting Information

Supporting Information

Supporting Information

Supporting Information

Supporting Information

## Data Availability

The TMT‐based and label‐free mass spectrometry proteomics data have been deposited in the ProteomeXchange Consortium (http://proteomecentral.proteomexchange.org) via the PRIDE^89^ and iProx^90^ partner repositories with the dataset identifiers PXD014994 and PXD022161, respectively. TMT‐based acetylated peptides and mass‐labeled MS/MS spectra have been uploaded to MS‐Viewer and the search key for the saved dataset is 6bblwp0gtv.
