# Supplementary figures and images for: Quantitative acetylome analysis reveals histone modifications that may predict prognosis in hepatitis B‐related hepatocellular carcinoma
Source: Clin Transl Med. 2021 Mar 8;11(3):e313. doi: 10.1002/ctm2.313 (PMC7939233; doi:10.1002/ctm2.313)

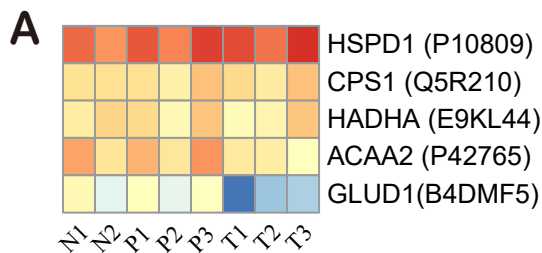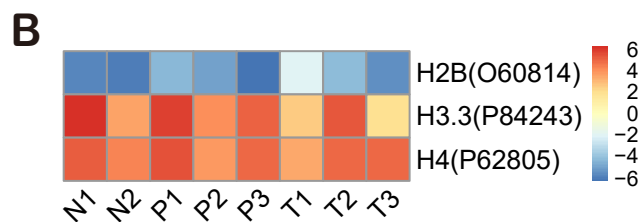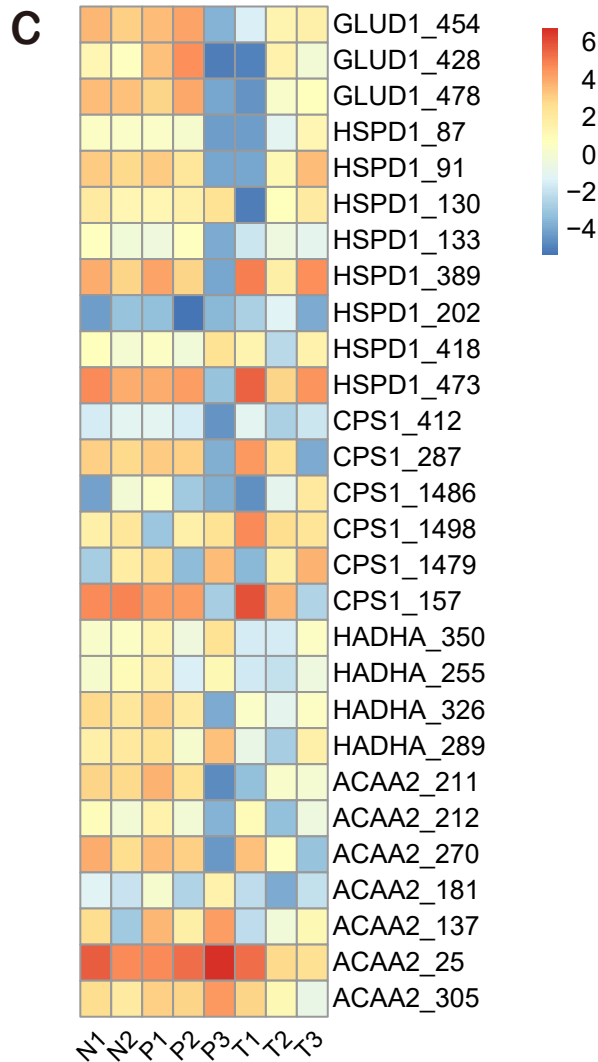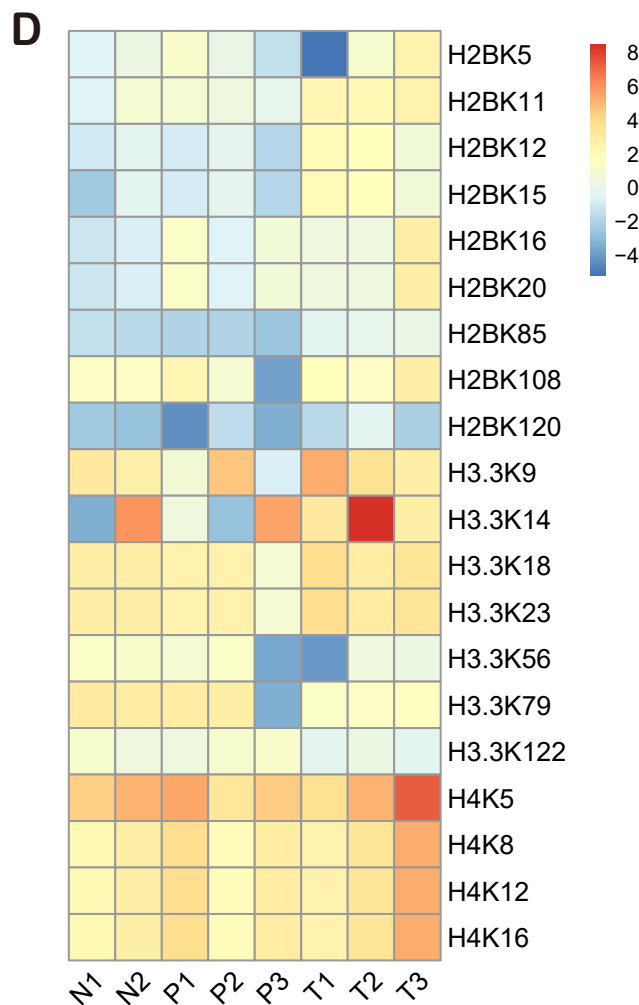

Supplement: Supplementary file 1 — Supporting Information [file CTM2-11-e313-s003.pdf]

**A**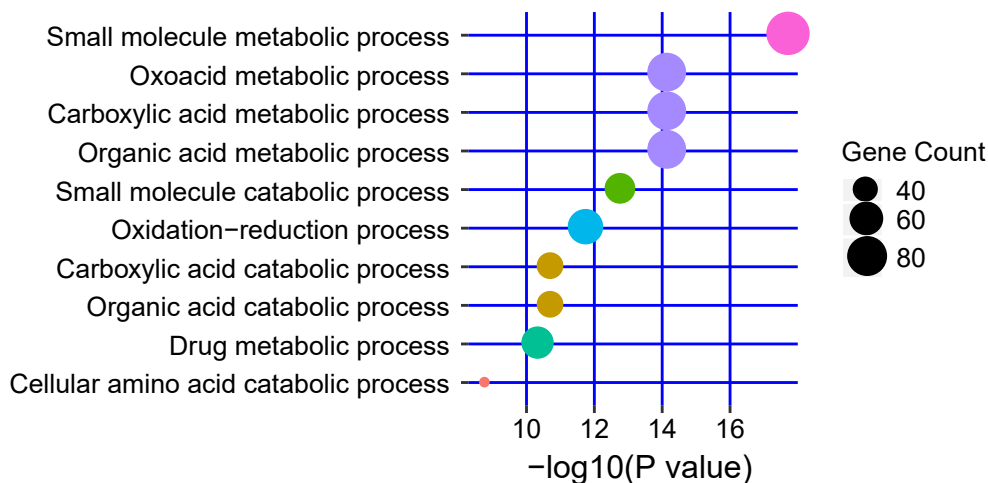**B**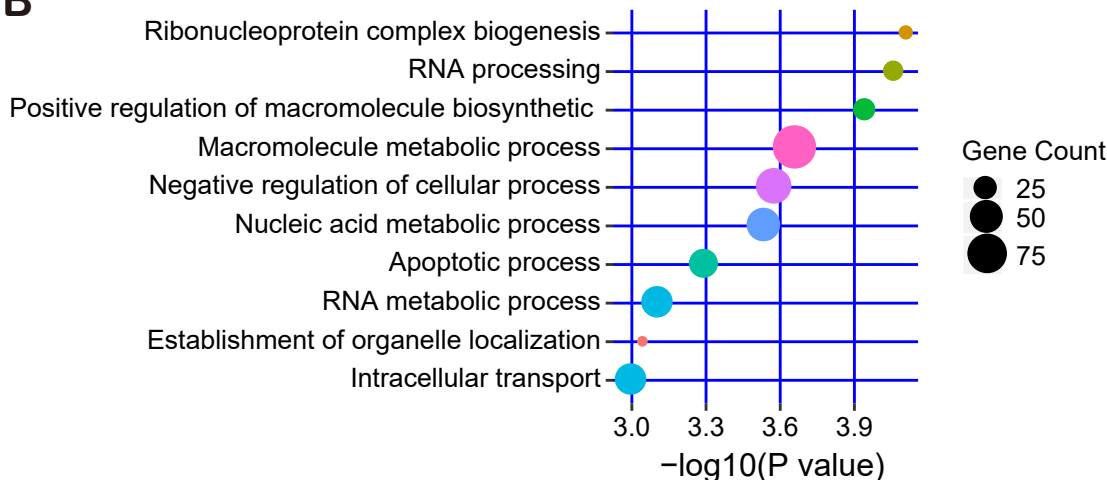

Supplement: Supplementary file 2 — Supporting Information [file CTM2-11-e313-s001.pdf]

**A**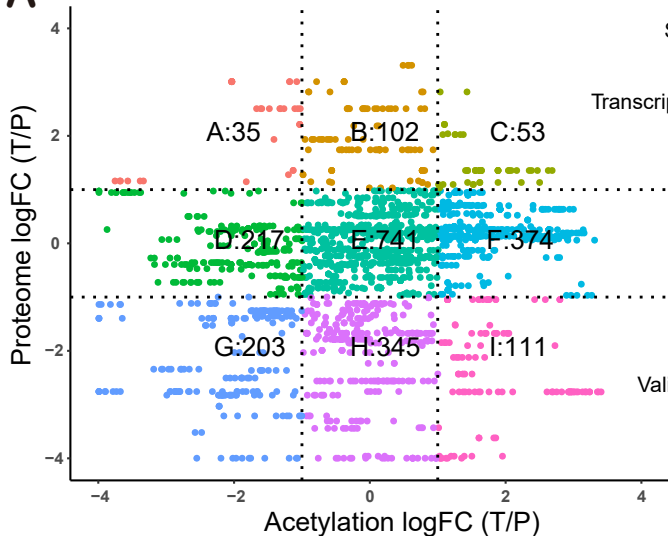**B**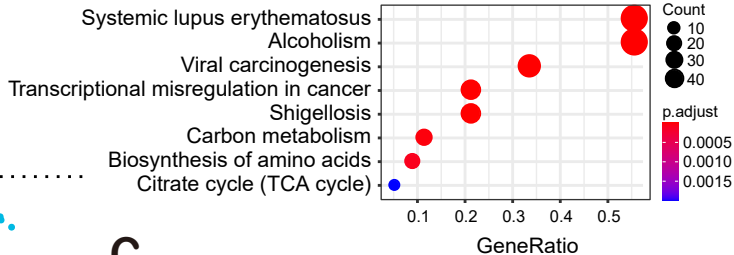**C**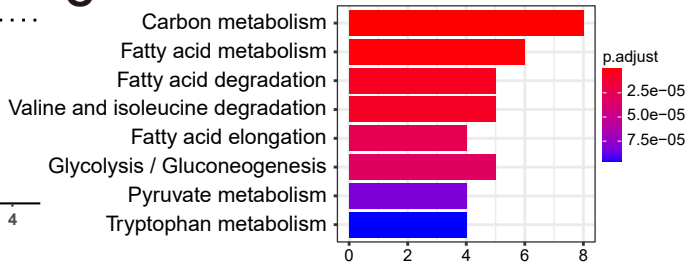

Supplement: Supplementary file 3 — Supporting Information [file CTM2-11-e313-s009.pdf]

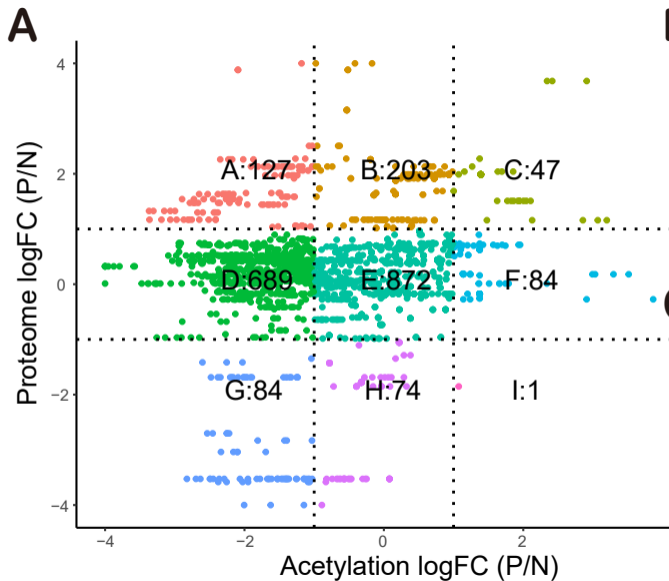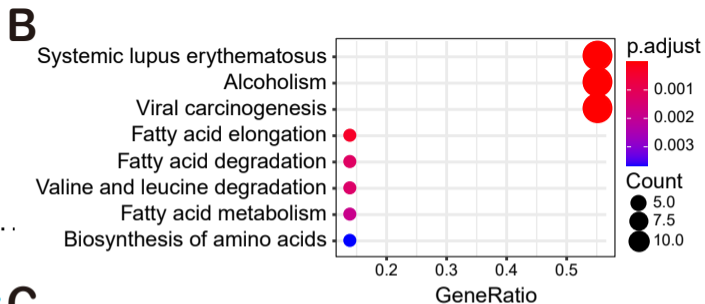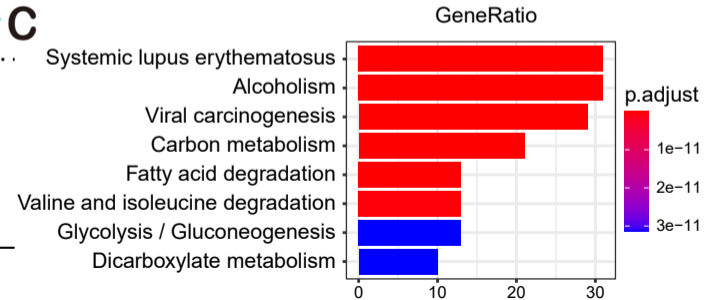

Supplement: Supplementary file 4 — Supporting Information [file CTM2-11-e313-s008.pdf]

**A**

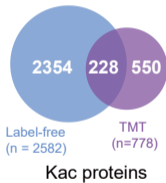

**B**

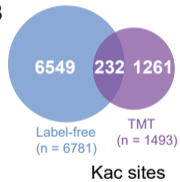

Supplement: Supplementary file 5 — Supporting Information [file CTM2-11-e313-s012.pdf]

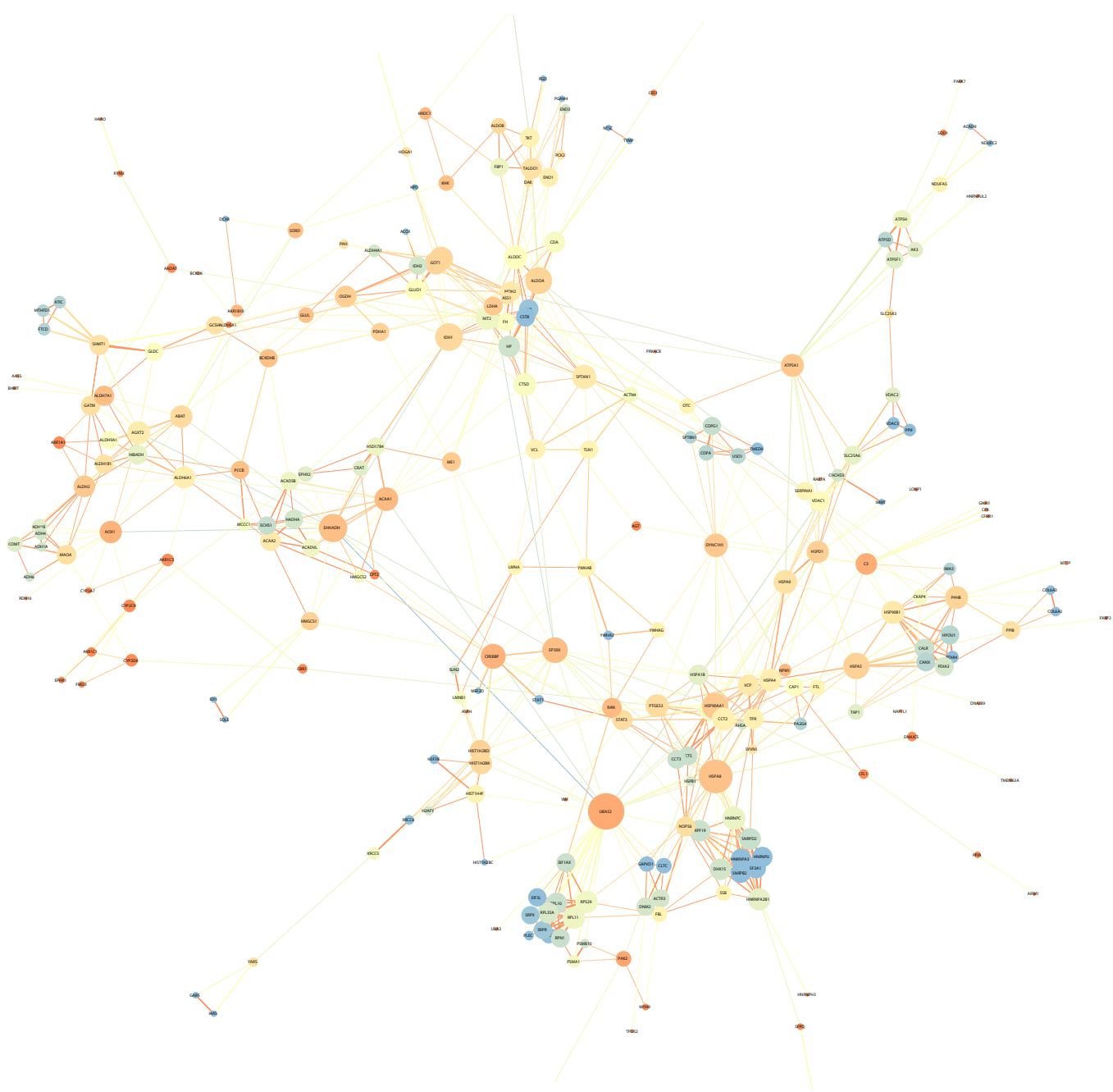

Supplement: Supplementary file 6 — Supporting Information [file CTM2-11-e313-s013.pdf]

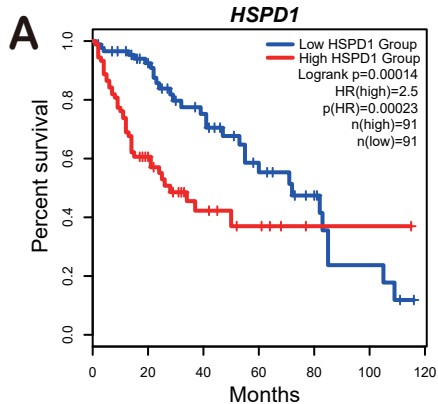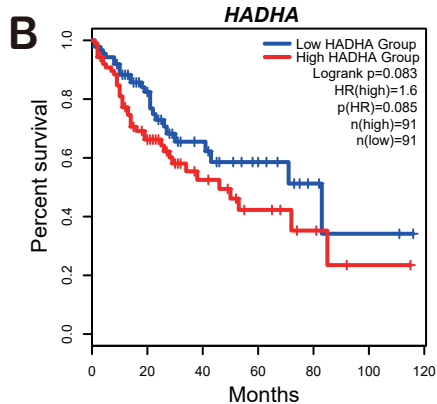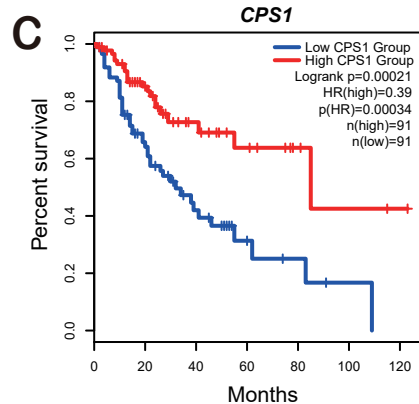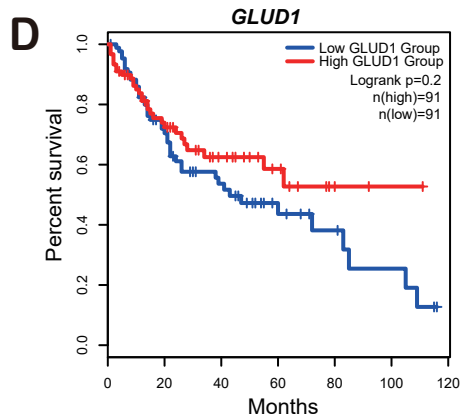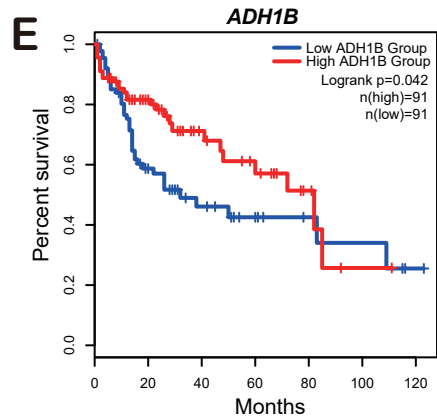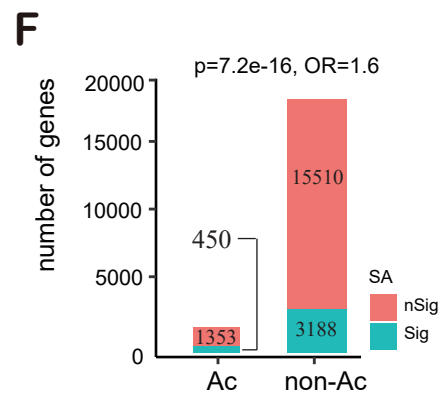

Supplement: Supplementary file 7 — Supporting Information [file CTM2-11-e313-s005.pdf]
